# Automated Test for Monthly Quality Assurance of Optical Surface Imaging Dynamic Localization Accuracy

**DOI:** 10.7759/cureus.56242

**Published:** 2024-03-15

**Authors:** Kaleigh Young, Eric A Wright

**Affiliations:** 1 Medical Physics, University of British Columbia - Okanagan Campus, Kelowna, CAN; 2 Medical Physics, Sunnybrook Health Sciences Centre Odette Cancer Centre, Toronto, CAN

**Keywords:** optical imaging, radiation therapy technology, optical surface imaging, surface-guided radiotherapy, general radiation oncology

## Abstract

The American Association of Physicists in Medicine (AAPM) recently published the report of Task Group (TG) 302, which provides recommendations on acceptance, commissioning, and ongoing routine quality assurance (QA) for surface-guided radiation therapy (SGRT) systems. One of the recommended monthly QA tests is a dynamic localization accuracy test. This work aimed to develop an automated procedure for monthly SGRT dynamic localization QA.

An anthropomorphic head phantom was rigidly attached to the 6-dof couch of a TrueBeam linac. TrueBeam Developer Mode was used to take an MV image of the phantom at the starting position, then automatically drive the couch through a series of translations and rotations, taking an MV image after each translation. The Identify SGRT system monitored the motion of the phantom surface from the starting position. Translations assessed on MV images were compared to translations reported in trajectory log files and Identify log files. Rotations were compared between trajectory log files and Identify log files.

Three experiments were conducted. None of the translations or rotations from any experiment exceeded the tolerance values for stereotactic ablative body radiation therapy (SABR) recommended by AAPM TG-142. Maximum deviations from the expected translation values from MV imaging, trajectory log files, and Identify log files were -0.94mm, -0.11mm, and -0.78mm, respectively. Maximum deviations from the expected rotation values from trajectory log files and Identify log files were 0.01 and -0.2 degrees, respectively. The proposed method is a simple automated way to complete monthly dynamic localization QA of SGRT systems.

## Introduction

Optical surface imaging systems for surface-guided radiation therapy (SGRT) have become increasingly common in North American cancer clinics in recent years. SGRT systems rely on ceiling-mounted camera-projector pods to provide quantitative, real-time patient positioning information using stereoscopic imaging of a speckled light pattern projected onto the patient [1]. The position and orientation of the patient surface are reported in 6 degrees of freedom (6-DoF) (i.e., vertical, longitudinal, and lateral translations, and rotations about each of the principal axes: rotation, roll, and pitch) relative to a reference position. In radiation therapy, treatments are typically planned on a CT scan and delivered using a linear accelerator over days to weeks. Therefore, reproducibility of patient positioning as closely as possible to their initial scanned position is important to ensure that treatment is delivered as planned. Patient setup is typically accomplished by aligning small marks tattooed on the patient’s skin to lasers in the treatment room. However, research has shown that SGRT systems can provide a more accurate setup than traditional methods for breast [2,3], lung [4], and prostate cancer treatments [5] without the need for tattoos [6,7]. Real-time, quantitative information from SGRT systems can also be used to ensure that the patient does not move away from the reference position during treatment delivery [8]. This allows treatments to be delivered with a high degree of precision while also using less restrictive immobilization devices since the SGRT system can detect unwanted intrafraction motion above a pre-specified threshold, prompting additional imaging and re-positioning, if necessary.

The American Association of Physicists in Medicine (AAPM) is a scientific and professional organization whose primary goal is identifying and implementing improvements in patient safety for the medical use of radiation in imaging and radiation therapy. The AAPM recently published the report of task group (TG) 302 [9], providing recommendations on acceptance, commissioning, and ongoing routine quality assurance (QA) for SGRT systems. The monthly QA outlined by this report includes checks of the safety systems, a static localization accuracy test, and a dynamic localization accuracy test. Safety systems vary depending on the SGRT system and are relatively straightforward, and static localization accuracy is typically assessed with a hidden target test using a vendor-supplied phantom and procedure. However, no vendor-supplied procedure exists for monthly QA of dynamic localization accuracy. Dynamic localization accuracy involves the ability of the SGRT system to accurately measure small displacements in real time. It can be tested by shifting a phantom by a known amount and determining the accuracy and temporal latency of the shift reported by the SGRT system. This is very important in the context of intra-fraction motion monitoring, particularly during stereotactic radiosurgery (SRS) treatments [10]. SRS treatments require a high degree of precision during planning and delivery to adequately treat brain metastases while limiting the volume of irradiated normal brain as much as possible to reduce the risk of symptomatic radiation necrosis [11,12]. A standard method for testing dynamic localization accuracy would help ensure that SGRT systems perform adequately for intra-fraction motion monitoring during treatment delivery. The objective of this work was to present a simple semi-automated procedure for monthly SGRT dynamic localization QA.

## Technical report

Methods

Phantom Setup

**Figure 1:**
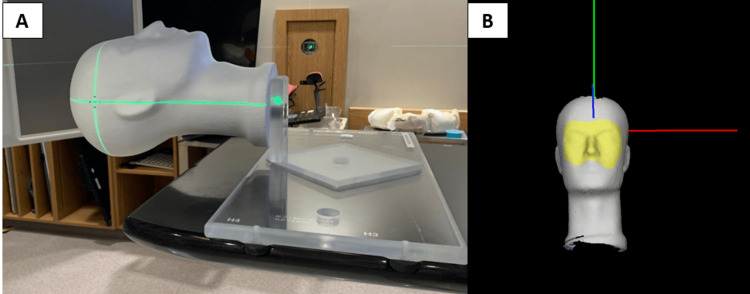
Anthropomorphic head phantom and jig Anthropomorphic head phantom and jig are set up on the linear accelerator couch (A), and the region of interest (yellow region) is used for monitoring with the Identify surface-guided radiation therapy system (B).

A 5 mm diameter metal bb was implanted inside of a Styrofoam head phantom. A jig was constructed to securely affix the phantom onto the 6-DoF couch (i.e., A couch capable of correcting patient position in 6-DoF by applying translations and rotations about the 3 principle axes) of a TrueBeam linear accelerator (Varian Medical Systems, Palo Alto, CA) (Figure 1A). TrueBeam linear accelerators are equipped with an X-ray detector panel mounted opposite the gantry head to facilitate X-ray imaging using the megavoltage (MV) treatment beam, commonly referred to as MV imaging. MV imaging was used to align the bb approximately at the isocentre. A reference surface was captured with the phantom in the starting position using Identify v2.2 (Varian Medical Systems, Palo Alto, CA) with a region of interest (ROI) covering from the eyebrows to below the nose (Figure 1B). The Identify ROI position and orientation were monitored in 6-DoF and recorded in a log file while the couch was translated and rotated using TrueBeam Developer Mode (Varian Medical Systems, Palo Alto, CA).

TrueBeam Developer Mode Scripting

TrueBeam Developer Mode is an application that allows couch, gantry, collimator, and MLC movements, as well as beam delivery and image acquisition, to be carried out by the linear accelerator based on XML scripting [13]. The XML script was designed to capture an MV image of the initial phantom position, then drive the couch through a list of translations and rotations, taking an MV image and delivering 50 MU after each translation. Briefly, the script translates the couch laterally and longitudinally by ±1cm and ±5cm. After the couch returns to the starting position, the gantry rotates to 90 degrees, and the couch is shifted vertically by ±1cm and ±5cm. The couch and gantry return to the starting position, and then the couch yaw, pitch, and roll are adjusted by ±3 degrees in 0.5-degree increments.

Data Analysis

Trajectory log files were recorded each time the developer mode script was run. Trajectory log files are text files that record the expected and actual status of linear accelerator parameters (e.g., position and orientation of 6-DoF couch) during beam delivery. The change in phantom surface position/angle was also recorded using Identify. Trajectory log files, Identify log files, and MV images were analyzed using MATLAB R2021b (Mathworks, Natick, MA). Identify log files were interpolated to the same temporal resolution as the trajectory log files, then synchronized by finding the first instance of motion in each log file and defining that as the start time. Couch position and rotation and Identify-reported phantom surface position and rotation were averaged over beam-on time at each couch position. The position of the bb center was automatically detected in an MV image at the starting position and in the MV images taken after each translation. Spatial offsets between the initial bb position, and the bb positions corresponding to each translation were determined for comparison with the couch translations reported in the trajectory log files and Identify log files.

Results

**Figure 2:**
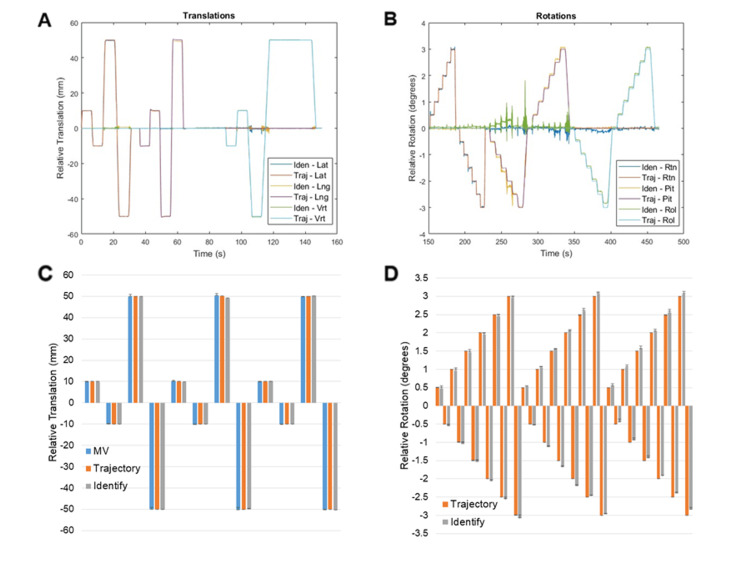
Identify and trajectory log file reported translations and rotations Synchronized translations (A) and rotations (B) from trajectory log files and Identify log files from each experiment. Median values from MV images (blue), trajectory log files (red), and Identify log files (grey) for each translation (C), and for each rotation (D). Error bars represent minimum and maximum values.

The script was run three times on a TrueBeam linear accelerator equipped with developer mode, a 6-DoF couch, and an Identify SGRT system. Translations and rotations were synchronized from trajectory log files and Identify log files, and results are summarized (Figure 2). Maximum deviations from the expected translation values from MV imaging, trajectory log files, and Identify log files were -0.94mm, -0.11mm, and -0.78mm, respectively. Maximum deviations from the expected rotation values from trajectory log files and Identify log files were 0.01 and -0.2 degrees, respectively.

## Discussion

This work presents a simple automated method for completing AAPM TG-302 compliant monthly dynamic localization QA of SGRT systems. Inserting a bb into the phantom and assessing couch translations with MV images provides an additional means of verifying true couch translations beyond trajectory log files. The largest deviations seen between the expected couch translations and couch translations assessed by MV imaging (-0.94mm) and Identify (-0.78mm) were below the tolerance values for SABR from AAPM TG-142 (1mm and 1 degree) [14], suggesting the precision of the SGRT system is sufficient for intrafraction motion monitoring during SABR and SRS procedures. Identify results showed excellent reproducibility, with results from all three experiments agreeing within 0.3 mm and 0.2 degrees. Results from trajectory log files also agreed within 0.2mm and 0.1 degrees across experiments. Translations measured on MV images agreed between the three experiments within 1mm. The reason for greater variability in translations assessed on MV images was likely caused by uncertainty in detecting the center of the bb in the images.

As SGRT continues to become more widely adopted, efficient and standardized QA methods will become more important. The procedure developed in this work can serve as a relatively simple, standardized method of assessing dynamic localization accuracy. The XML script used for Varian Developer Mode and the Matlab script for data analysis can be made available upon request to the corresponding author.

There are two limitations to the current procedure. Firstly, couch yaw, pitch, and roll are assessed only with Identify and not with MV images because only a single bb was included in the phantom, making it impractical to determine rotations from MV images. Future iterations of the phantom can include multiple bbs or wires to facilitate radiographic verification of couch rotations. Secondly, part of dynamic localization QA for SGRT systems includes quantitative assessment of temporal latency. The current procedure is limited to qualitative assessment of the alignment of trajectory log files and Identify log files. Quantitative assessment of this alignment holds little value because of how the files are aligned based on the first instance of motion (i.e., any time lag seen between log files is likely due to misalignment of the two log files by the MATLAB script and not actual temporal latency). In Identify v2.2, log files do not contain a beam-on flag, but other SGRT vendors and newer versions of Identify will provide this information in the log files. In the future, the script will align log files from the first instance of beam-on, then search for the time lag between motion traces in the trajectory log and Identify log files, providing a quantitative analysis of temporal latency.

## Conclusions

A simple procedure for semi-automated monthly dynamic localization QA of SGRT systems was developed. The procedure uses an inexpensive, widely available styrofoam anthropomorphic head phantom with an implanted bb.
